# How to Change the Oligomeric State of a Circular Protein Assembly: Switch from 11-Subunit to 12-Subunit TRAP Suggests a General Mechanism

**DOI:** 10.1371/journal.pone.0025296

**Published:** 2011-10-03

**Authors:** Chao-Sheng Chen, Callum Smits, Guy G. Dodson, Mikhail B. Shevtsov, Natalie Merlino, Paul Gollnick, Alfred A. Antson

**Affiliations:** 1 York Structural Biology Laboratory, Department of Chemistry, University of York, York, United Kingdom; 2 Biological Sciences, University of Portsmouth, King Henry Building, Portsmouth, United Kingdom; 3 Department of Biological Sciences, State University of New York at Buffalo, Buffalo, New York, United States of America; Russian Academy of Sciences, Institute for Biological Instrumentation, Russian Federation

## Abstract

**Background:**

Many critical cellular functions are performed by multisubunit circular protein oligomers whose internal geometry has evolved to meet functional requirements. The subunit number is arguably the most critical parameter of a circular protein assembly, affecting the internal and external diameters of the assembly and often impacting on the protein's function. Although accurate structural information has been obtained for several circular proteins, a lack of accurate information on alternative oligomeric states has prevented engineering such transitions. In this study we used the bacterial transcription regulator TRAP as a model system to investigate the features that define the oligomeric state of a circular protein and to question how the subunit number could be manipulated.

**Methodology/Principal Findings:**

We find that while *Bacillus subtilis* and *Bacillus stearothermophilus* TRAP form 11-subunit oligomers, the *Bacillus halodurans* TRAP exclusively forms 12-subunit assemblies. Significantly, the two states of TRAP are related by a simple rigid body rotation of individual subunits around inter-subunit axes. We tested if such a rotation could be induced by insertion or deletion mutations at the subunit interface. Using wild type 11-subunit TRAP, we demonstrate that removal of five C-terminal residues at the outer side of the inter-subunit axis or extension of an amino acid side chain at the opposite, inner side, increased the subunit number from 11 to 12. Our findings are supported by crystal structures of TRAP oligomers and by native mass spectrometry data.

**Conclusions/Significance:**

The subunit number of the TRAP oligomer can be manipulated by introducing deletion or addition mutations at the subunit interface. An analysis of available and emerging structural data on alternative oligomeric states indicates that the same principles may also apply to the subunit number of other circular assemblies suggesting that the deletion/addition approach could be used generally to engineer transitions between different oligomeric states.

## Introduction

Multisubunit circular proteins play key roles in a variety of biological mechanisms. A number of proteins including bacterial toxins [1], viral portal proteins [2], flagellar motor proteins [3], [4], Sm-like proteins [5], components of the type III secretion system [6] and protective antigen of anthrax toxin [7], [8] can form circular oligomers with alternative oligomeric states. In some cases, for example for the viral portal proteins, a single functional oligomeric state is found *in vivo* and it is not known if the alternative oligomeric states are active. However, in all cases changing the number of constituents in the ring will alter the symmetry, the internal cavity diameter and curvature of the protein ring, these changes may be critical to the function. Here we examined the factors that define the oligomeric state in a circular protein oligomer by studying the *Bacillus trp* RNA-binding attenuation protein (TRAP).

TRAP regulates transcription and translation of tryptophan biosynthetic genes in many *Bacilli*, including *Bacillus subtilis*, *Bacillus pumilus*, *Bacillus stearothermophilus*, *Bacillus licheniformis* and *Bacillus halodurans* [9], [10]. Studies on *B. subtilis* have shown that when TRAP is activated by bound tryptophan, it can recognize and bind specific segments of RNA [11], [12]. When the tryptophan concentration is high transcription attenuation occurs as TRAP, binding to the leader RNA region of the *trpEDCFBA* operon transcript, induces the formation of a transcription terminator hairpin thus preventing the establishment of an antiterminator structure. When the concentration of tryptophan is limiting, TRAP is inactive, which allows transcription to proceed into the structural genes [13].

Previously determined X-ray structures of *B. subtilis* and *B. stearothermophilus* TRAP [14], [15] revealed 11-subunit oligomers with essentially identical architecture. In most *Bacillus* species the *trp* leader segment that interacts with TRAP contains 11 NAG triplets (N is predominantly G or U) separated by two or three spacer nucleotides [15]. The X-ray structure of *B. stearothermophilus* TRAP in complex with RNA showed how the 11 triplets of RNA are matched by 11 binding sites generated at the outer surface of the 11-subunit TRAP [16]. Unlike most other *Bacillus* species, the RNA leader region in the alkaliphilic bacterium *B. halodurans* contains 19 NAG triplets, which could potentially interact with TRAP. The oligomeric state of *B. halodurans* TRAP was unknown and we speculated that it might contain more than eleven subunits.

**Figure 1:**
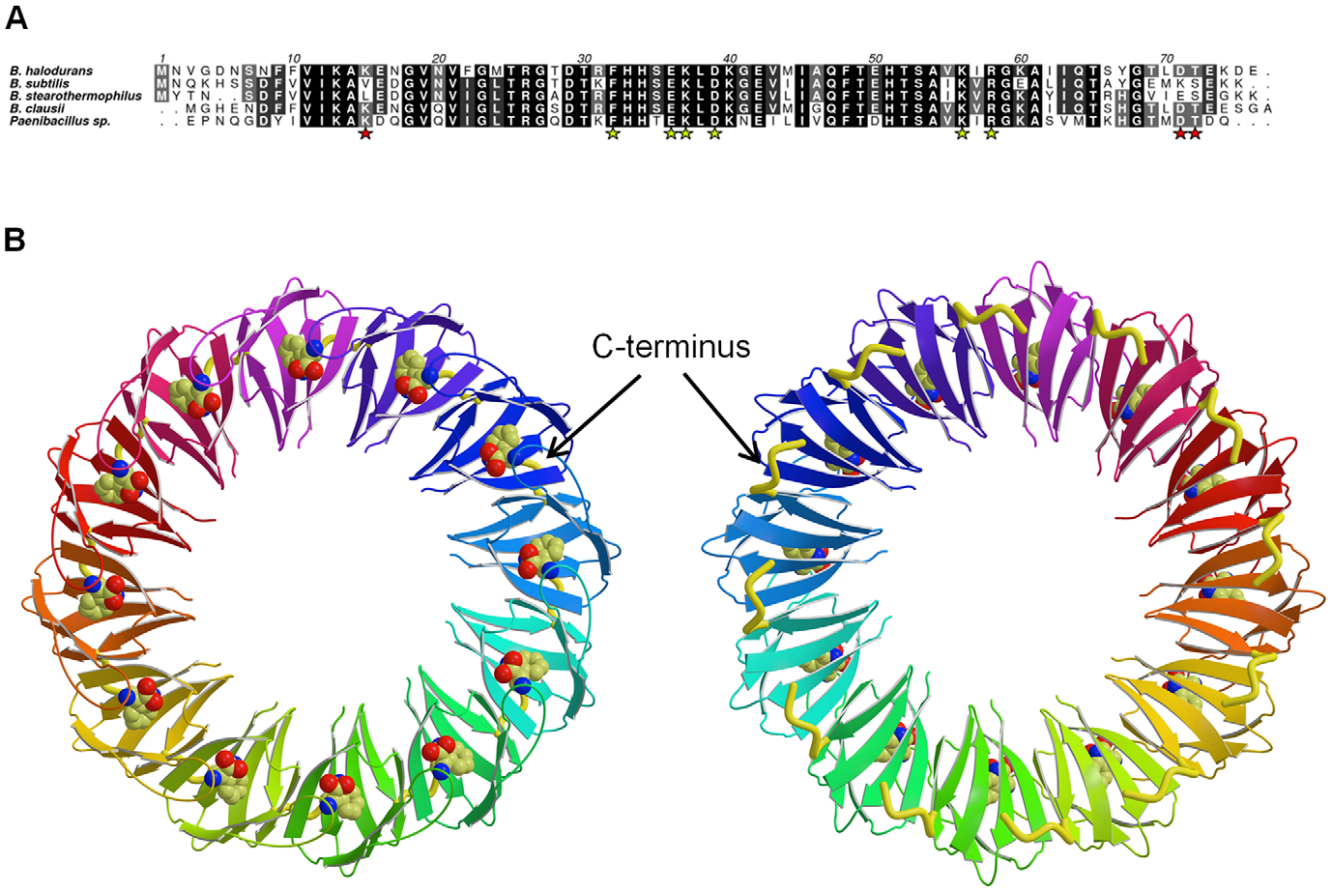
Structure of *B. halodurans* TRAP. (A) Sequence alignment of TRAP proteins from different species produced by ClustalW [18]. The extent of sequence conservation is depicted in grey with identical residues in black and non-conserved in white. Residues important for the conformation of the C-terminus are highlighted by red stars and residues that interact with RNA are highlighted by yellow stars. (B) Ribbon diagrams viewed along the 12-fold axis (two opposite views). Each subunit is shown in a different color. The C-terminus of each subunit, starting from residue 71, is shown by a thick yellow ribbon. L-Tryptophan molecules, bound in deep pockets between adjacent subunits, are shown as van der Waals models with carboxyl oxygen atoms in red, nitrogen atoms in blue and carbon atoms in yellow.

*B. halodurans* grows at pH values above 9.5 and contains a 76 amino acid TRAP protein [17] that shares ∼71% sequence identity with *B. subtilis* TRAP, **Figure 1A**. Like *B. subtilis* TRAP, *B. halodurans* TRAP has a high affinity towards the *trp* leader RNA [10]. We characterized *B. halodurans* TRAP by native mass spectrometry and determined its crystal structure at 1.7 Å resolution. We show that unlike the 11-subunit TRAP found in *B. subtilis* and *B. stearothermophilus*, *B. halodurans* TRAP is a 12-subunit oligomer, implying that this larger oligomeric state has some functional benefit. The most significant difference between the structures is a conformation change at the C-terminus. This change alters the interface interactions and we hypothesized that these changes were responsible for the change in oligomeric state. We tested this hypothesis by creating mutant *B. subtilis* and *B. stearothermophilus* TRAP proteins in which the last five amino acids were removed. X-ray structures of these engineered TRAP, reported here at a high resolution, together with the native mass spectrometry data, show that as predicted, these truncated proteins exist as 12-subunit assemblies. Notably, with the exception of the C-terminus, the 11-mer to 12-mer transition is not accompanied by any significant conformational changes within individual subunits. The 12-mer state is generated from the 11-mer state by a simple rigid-body rotation of individual subunits around inter-subunit axes, triggered by the amino acid deletion at one side of the axis. Each axis crosses a central part of the subunit-subunit interface and is roughly parallel to the tunnel axis. We tested if the 11-mer to 12-mer transition could be also induced by an addition at the opposite, inner side of the axis. We mutated Val11 to Leu in *B.subtilis* TRAP and obtained native mass spectrometry data, showing that this mutation also results in 11-mer to 12-mer transition. We argue that similar approach could be used for engineering a transition between different states of other circular proteins.

## Results

### Crystal structure of *B. halodurans* TRAP

*B. halodurans* TRAP (PDB accession code 3ZZL) forms a circular assembly containing 12-subunits, **Figure 1B**. The 12-mer in the crystal is generated by combination of three subunits (present in the asymmetric unit) with the crystallographic 4-fold axis of the P42_1_2 space group. The structure was refined with data extending to 1.67 Å, **Table 1**. The final electron density maps allowed the positioning of all residues, except the six N-terminal residues of all subunits. The diameter of the central tunnel, measured for the Cα positions of Ser7, is 30.7 Å, an increase from 26.6 Å observed in the *B. stearothermophilus* TRAP. Like the 11-mer TRAP molecule, the 12-mer assembly of *B. halodurans* TRAP is stabilized by an identical pattern of main chain - main chain hydrogen bonding interactions, **Table S1**. These interactions link a three-stranded β-sheet from one subunit with a four-stranded β-sheet from the adjacent subunit resulting in the formation of twelve seven-stranded inter-subunit β-sheets per oligomer.

**Table 1:** Data collection and refinement statistics.

	*B. halodurans* TRAP	*B. stearo* E71stop TRAP	*B. subtilis* K71stop TRAP
Data collection			
Space group	P4212	I4	I23
Unit cell	*a* = 109.9 Å, *c* = 45.7 Å	*a* = 110.2 Å, *c* = 128.3 Å	*a* = 146.35 Å
Resolution	30–1.67 Å (1.73–1.67 Å)	25–1.49 Å (1.52–1.49 Å)	25–1.75 Å (1.79–1.75 Å)
No. of reflections	32619 (2996)	124338 (6184)	51357 (3760)
Redundancy	8.3 (6.2)	4.9 (3.7)	25.4 (22.3)
*R*_merge_ a, %	9.1 (63.8)	4.7 (48.1)	8.9 (58.7)
Completeness, %	98.6 (92.8)	99.9 (100)	99.9 (100)
I/σ	19.4 (2.0)	31.5 (2.7)	42.9 (6.3)
Wilson B factor	26.8	16.8	23.9

Refinement			
Resolution rang	30–1.67 Å (1.71–1.67 Å)	25–1.49 Å (1.52–1.49 Å)	25–1.75 Å (1.79–1.75 Å)
Number of reflections	31492	12301	51357
used in refinement			
Number of reflections	1038	1250	1030
excluded from refinement			
*R* factor^b^, %	18.0 (27.4)	14.7 (21.8)	17.3 (19.2)
Free *R* factor^b^, %	21.5 (27.7)	18.3 (26.3)	19.0 (20.3)
Number of atoms			
Protein	1977	5950	3129
Ligand	90	135	180
Water	226	962	308
Average B factor (protein)	27.3	14.1	22.6
Average B factor (solvent)	49.1	42.5	40.4
Rmsd bond lengths	0.010 Å	0.007 Å	0.007 Å
Rmsd bond angles	1.2°	1.1°	1.1°
Ramachandran plot			
Most favored regions, %	100	99.6	99.4
Additionally allowed	0	0.4	0.6
regions, %			

Values in parentheses are for the highest resolution shell.

a *R_merge_*  =  ∑*_hkl_*∑_i_|*I_i_(h)* - <*I(h)>*|/∑*_hkl_*∑_i_ *I_i_(h)*, where *I(h)* is intensity of reflection *h*, <*I(h)>* is average value of intensity, the sum ∑*_hkl_* is over all measured reflections and the sum ∑_i_ is over *i* measurements of a reflection.

b Crystallographic *R  = * ∑*_hkl_*||*F_obs_* - *F_calc_*||/∑*_hkl_*|*F_obs_|*, *R_free_* was calculated using a randomly chosen set of reflections that were excluded from the refinement.

### Both 11-subunit and 12-subunit TRAP assemblies exist in nature

The crystallographic and mass spectrometry data reported here show that *B. halodurans* TRAP exists as 12-subunit oligomer, **Figure 1B** **and** **Table 2**. Thus, both the eleven and twelve subunit assemblies have been selected by different species to perform the same function. It is possible that the increase to 12 subunits is favored by the apparent redundancy in triplet repeats in *B. halodurans* [10]. In spite of the difference in the number of subunits and the angular separation between individual subunits, the affinity of the *B. halodurans* TRAP 12-mer towards the *trp* leader RNA is essentially the same as for *B. subtilis* TRAP 11-mer, with the *K_d_* being ∼4 nM in each case [10]. The similar affinities are generated because of conservation of the RNA-binding residues leading to similar binding motifs, **Figure 1A**, and by comparable distances between adjacent NAG-binding pockets on the protein's surface, **Table 3**.

**Table 2:** Summary of native mass spectrometry analysis.

Oligomer	Calculated	Measured	Error [measured – calculated]
	mass, Da	mass, Da	mass, Da (%)
*B. halodurans*			
TRAP_12_:trp_12_	104361.0	104399.9±11.0	38.9 (0.04)
TRAP_12_:trp_11_	104156.8	104188.7±1.2	31.9 (0.03)
TRAP_12_:trp_10_	103952.5	103985.1±0.2	32.6 (0.03)
TRAP_12_:trp_9_	103748.3	103773.3±1.4	25.0 (0.02)
*B. stearothermophilus*			
Wild type			
TRAP_11_:trp_11_	92912.5	93051.4±12.5	138.9 (0.15)
*B. stearothermophilus*			
E71stop mutant			
TRAP_12_:trp_12_	95004.0	95552.1±16.5	548.1 (0.58)
TRAP_12_:trp_11_	94799.8	94884.8±16.5	84.0 (0.09)
TRAP_12_:trp_10_	94595.5	94674.1±11.3	78.6 (0.08)
TRAP_12_:trp_9_	94391.3	94479.2±13.6	87.9 (0.09)
TRAP_12_:trp_8_	94187.0	94279.4±5.2	92.4 (0.10)
TRAP_12_:trp_7_	93982.9	94088.2±11.0	106.2 (0.11)
TRAP_12_:trp_6_	93778.6	93878.2±17.4	99.6 (0.11)
*B. subtilis*			
V11L mutant			
TRAP_12_:trp_3_	100722.6	100844.09±0.0	121.49 (0.12)
TRAP_12_:trp_3_	100722.6	100877.99±0.8	155.39 (0.15)

The oligomeric states of TRAP and numbers of bound tryptophan molecules are indicated in the left column.

**Table 3:** Average distance (Å) between Cα atoms of RNA-binding residues belonging to adjacent subunits.

Amino acid	*B. subtilis*	*B. stearothermophilus*	*B. halodurans*
	TRAP	TRAP	TRAP
F32	18.5	18.5	18.0
E36	18.7	18.9	18.4
K37	20.2	20.3	19.6
D39	20.7	20.9	20.2
K56	16.4	16.7	16.4
R58	19.0	19.3	18.7

Amino acid numbering corresponds to *B. subtilis* TRAP.

### Rotation of subunits during the 11-mer to 12-mer switch

**Figure 2:**
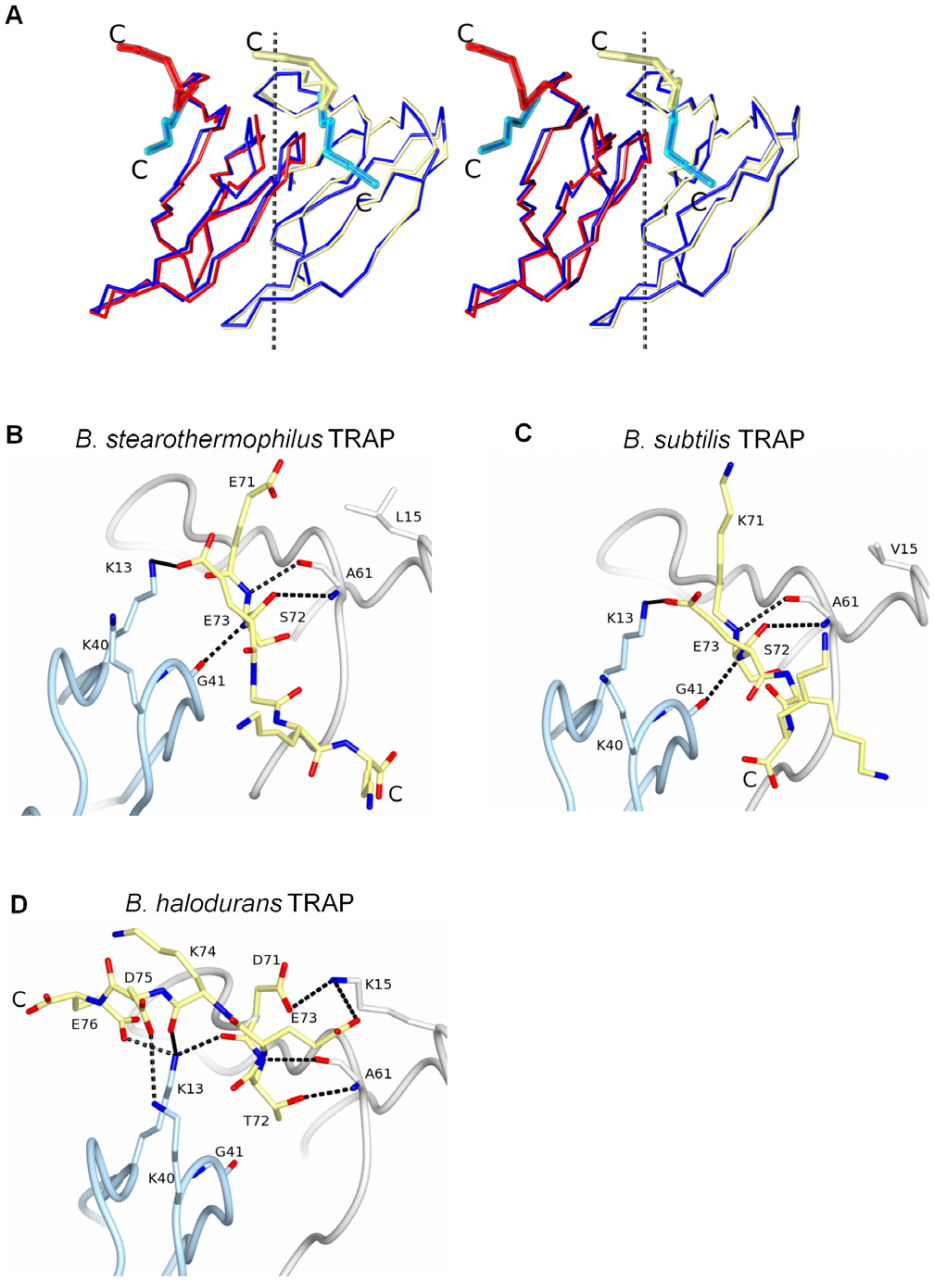
Comparison of 11-mer and 12-mer TRAP assemblies. (A) Dimers of *B. halodurans* TRAP (red and yellow) and wild-type *B. stearothermophilus* TRAP (both subunits in blue) were least-square fitted using main chain atoms of single subunit (shown on the right). Cα-models are shown with the segment 72–75 highlighted by wide traces. View is from outside the TRAP ring toward its center with the central rotation axis vertical. The inter-subunit rotational axis relating the 11-mer and 12-mer oligomers is shown by dashed line. (B, C, and D) Comparison of the C-terminus conformation in 11-mer and 12-mer TRAP. C-terminal residues starting from 71 and residues stabilizing the conformation of the C-terminus are shown by sticks, the rest of the subunit interface is shown by ribbons.

The main chain r.m.s. difference between residues 8–70 of *B. stearothermophilus* TRAP 11-mer (wild type) and *B. halodurans* TRAP 12-mer is 0.48 Å, indicating that the 11-mer to 12-mer transition is not accompanied by significant conformational changes in the protein. The transition is achieved largely by the 2.7° rigid-body rotation of individual subunits around inter-subunit axes. These axes, relating rotational adjustments between adjacent subunits, **Figures 2A** **and** **3**, are roughly parallel to the tunnel axis. Each axis crosses a central part of the subunit-subunit interface thus minimizing structural changes at the interfaces during the rotation. The 2.7° rigid-body rotation of individual subunits is accompanied by much smaller positional adjustments in individual residues easily accommodated by the plasticity in the protein's structure. These adjustments serve to maintain the interface and its individual contacts while allowing the overall rotation of subunits with respect to each other. As a result, there are only subtle changes in subunit-subunit interactions. For example, the lengths of the inter-subunit main chain hydrogen bonds that link β-strands belonging to adjacent subunits are very similar in the 12-mer and 11-mer TRAP proteins, **Table S1**, although the differences increase with greater distance from the inter-subunit axis.

**Figure 3:**
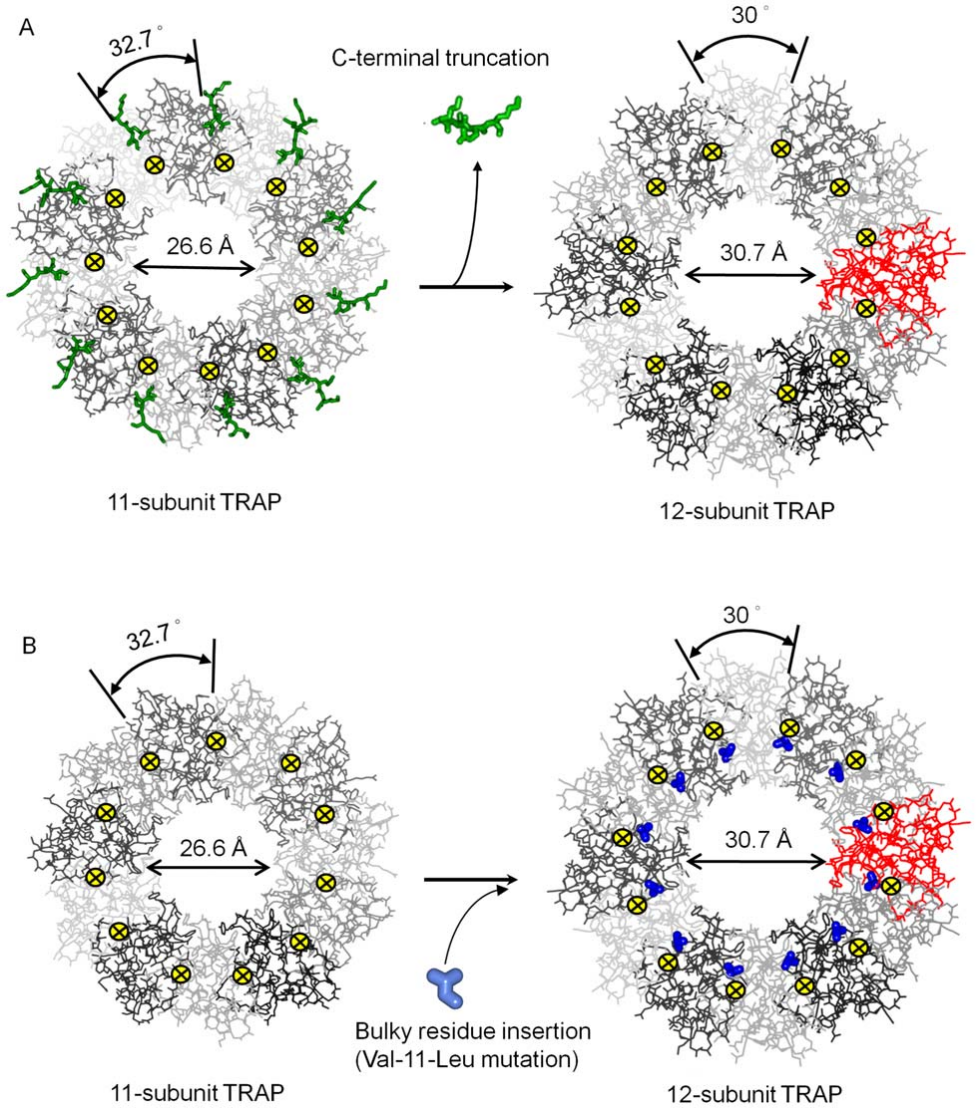
11-mer to 12-mer transition. Individual TRAP subunits are shown as ball-and-stick models in different shades of grey. Inter-subunit rotation axes are roughly parallel to the central oligomer axis and are depicted by black crosses shown in yellow circles. (A) Removal of the C-terminal segment (green) at one side of the axis or (B) introduction of methylene group through Val-11-Leu mutation (blue) at the other side of the axis, allows subunits to roll around the inter-subunit rotation axis to form a 12-mer.

### Role of C-terminal residues in selecting between different oligomeric states

The 2.7° rotation of neighboring subunits with respect to each other is possible owing to a significant change in the conformation of the five C-terminal amino acids in *B. halodurans* TRAP. In both *B. subtilis* and *B. stearothermophilus* TRAP, Ser72 and Glu73 are positioned at the subunit-subunit interface, **Figures 2B, C**. Ser72 is part of a β-strand, making two main chain – main chain hydrogen bonding interactions with Ala61 from the adjacent β-strand of the same subunit. Glu73 also has the β-strand conformation but its main-chain atoms are exposed towards the adjacent subunit resulting in an inter-subunit main chain hydrogen bonding interaction formed by its nitrogen atom with the carbonyl oxygen of Gly41. In *B. halodurans* TRAP Thr72 and Glu73 have different conformations being displaced from the interface towards the outer surface of the molecule, **Figure 2D**. This results in the rupture of the main chain - main chain hydrogen bond between the conserved residues, Gly41 and Glu73. The different conformation of the C-terminal residues is stabilized by a new set of salt bridges formed by the pairs of residues Glu73/Lys15 and Glu76/Lys13, **Figure 2D**. Stabilizing intersubunit hydrogen bonding interactions are also formed by the side chain of Lys13 with main chain carbonyls of Asp71 and Lys74. In addition, most interfaces (8 out of 12) contain a salt bridge between Asp75 and Lys40. The displacement of the C-terminal residues from the subunit-subunit interface observed in *B. halodurans* TRAP hinted at the possibility that the C-terminus plays a key role in selecting between the 11-mer and 12-mer assemblies.

### Design of 12-mer TRAP by deletion or addition at subunit interface

We hypothesized that the 11-mer to 12-mer switch could be induced by withdrawal of the C-terminal amino acids from the subunit-subunit interface, as observed in *B. halodurans* TRAP. Such a switch could be engineered by simply removing the C-terminal amino acids starting from position 72. We tested this idea using *B. stearothermophilus* TRAP which normally forms 11-mers. The E71stop *B. stearothermophilus* TRAP mutant protein was truncated after residue 71 thus removing the five C-terminal amino acids and the main chain hydrogen bond between Gly41 and Glu73. The crystal structure of E71stop TRAP (PDB accession code 3ZZS), refined with data extending to 1.49 Å, **Table 1**, shows that this engineered protein indeed forms 12-mers, **Figure S1**. Likewise, removing the last four amino acids from the C-terminus of *B. subtilis* TRAP, which is shorter by one amino acid compared to the *B. stearothermophilus* TRAP (**Figure 1A**), also generated a 12-subunit oligomer, **Figure S1**. The structure of this mutant *B. subtilis* TRAP (PDB accession code 3ZZQ) was refined with data extending to 1.75 Å, **Table 1**.

Both *B. subtilis and B. stearothermophilus* engineered 12-mer TRAP molecules have overall architectures almost identical to that of the *B. halodurans* 12-mer TRAP, **Figure S1**. The conformation of individual subunits is also very similar, with the main chain r.m.s. difference with the *B. halodrurans* TRAP (residues 8–65) of 0.38 Å and 0.33 Å, respectively, for the engineered *B. stearothermophilus* and *B. subtilis* TRAP. The engineered 12-mer TRAP share almost identical intersubunit hydrogen bonding patterns with those in wild type 11-mers, **Tables S2 and S3**. All TRAP molecules analyzed have 5 equivalent main-chain hydrogen bonding pairs generated between the two β-strands belonging to adjacent subunits, **Table S1**. All 12-mer TRAP proteins bind tryptophan molecules in essentially identical manner as 11-mer TRAP, **Text S1** and **Figure S2**.

We hypothesized that the rotational adjustment of subunits, similar to that generated by deletion at the outer side of the intersubunit axis, **Figure 3A**, could be also achieved by addition at its inner side, as shown on **Figure 3B**. We tested this hypothesis by introducing a slightly bulkier leucine residue in place of valine (V11L mutation) in *B. subtilis* TRAP. Although we were not able to crystallize this mutant TRAP, the mass spectrometry data presented in the next section clearly demonstrate that unlike the wild type 11-mer, this mutant TRAP also forms 12-mers, as predicted.

### Native mass spectrometry of TRAP

**Figure 4:**
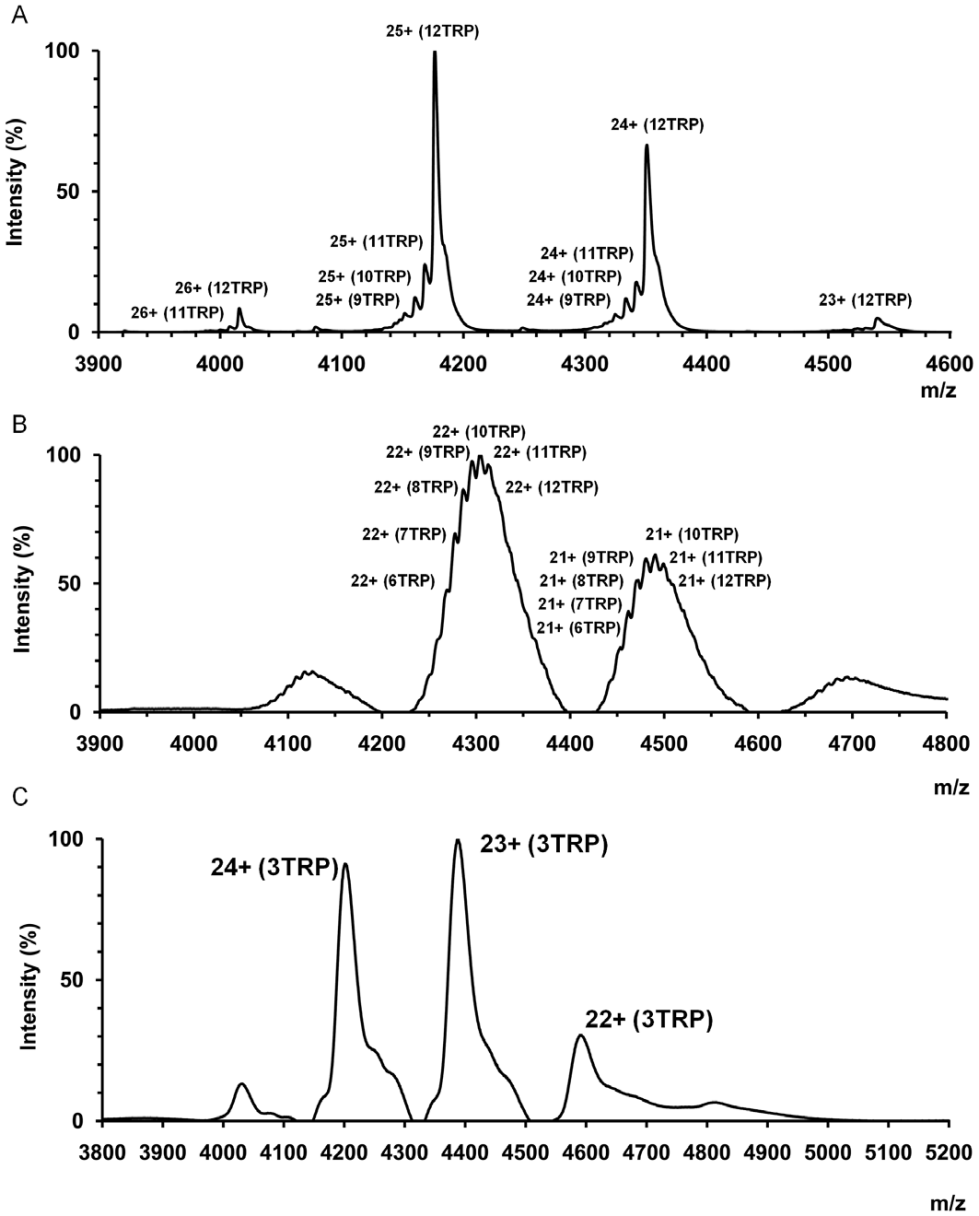
Native mass spectrometry analysis. Nanoflow electrospray mass spectra for (A) *B. halodurans* TRAP, (B) *B. stearothermophilus* E71stop TRAP and (C) *B. subtilis* V11L TRAP. A stable 12-mer species was identified in each sample; peaks corresponding to different charge states and different numbers of bound tryptophan molecules are labeled.

Previous analysis of *B. subtilis* TRAP by native mass spectrometry showed that its preferred oligomeric state is an 11-mer [19], [20] as found in the crystal structure [14]. We questioned whether the 12-subunit assemblies of *B. halodurans* TRAP and *B. stearothermophilus* E71stop TRAP predominate in solution or whether these were selected from a mixture of different oligomeric states during crystallization. For *B. halodurans* TRAP, the single stable species identified from mass spectra is the 12-mer, **Figure 4A** and **Table 2**. In addition, 12-mers with different number of bound L-tryptophan molecules were observed with maximum of 12 tryptophans per oligomer. For the *B. stearothermophilus* E71stop TRAP, again only one stable species was detected during the mass-spectrometry experiments, which corresponded to the 12-mer assembly. For this mutant TRAP, states with different number of bound tryptophan molecules were resolved in the presence of 1 µM L-tryptophan, **Figure 4B**. In the case of the *B. subtilis* V11L TRAP, the single 12-mer specie has been detected during the experiment, although for this mutant it was not possible to resolve states corresponding to different number of bound tryptophan molecules, **Figure 4C**. Control spectra obtained for *B. stearothermophilus* TRAP correspond to the 11-subunit oligomer, **Table 2**.

## Discussion

### Structural reorganization during the transition between different oligomeric states

Several proteins found in nature can form alternative circular assemblies. Sm and Sm-like proteins involved in nucleic acid processing were found as hexamers, heptamers and octamers [21], [22]. Two states corresponding to a hexamer and heptamer were resolved for the AAA+ domain of the NtrC-like transcriptional regulators [23], [24]. Bacteriophage SPP1 portal protein was found as a 12-mer assembly in mature capsids but forms non-functional 13-subunit assemblies when over expressed [2]. In this report we show that *B. halodurans* TRAP forms 12-subunit assemblies, unlike the *B. subtilis* and *B. stearothermophilus* TRAP that form 11-subunit oligomers. If like in TRAP, the functional interactions are at the surface and do not involve the central tunnel, it is clear that the same function can be accomplished by different oligomeric states. These structures also suggest that the oligomeric state could be manipulated so that the protein's properties are optimized for the required function.

**Figure 5:**
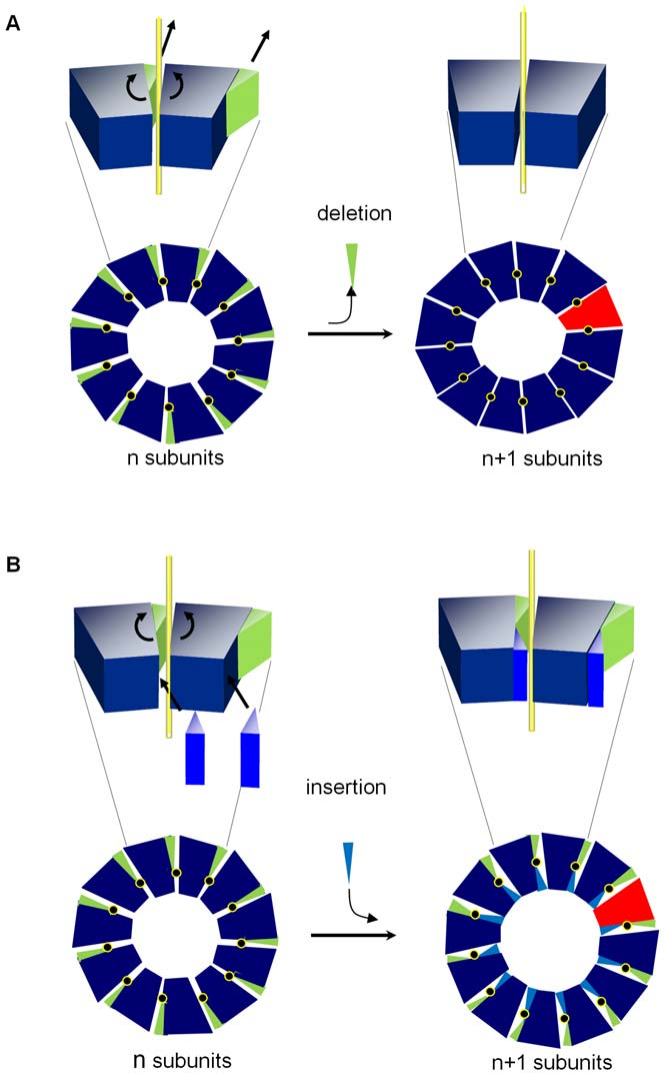
General model for transition between alternative oligomeric states. Transition from n-subunit to (n+1)-subunit state could be accomplished either by deletion (green triangles) at the outer side of the inter-subunit rotation axis (A) or insertion (light blue triangles) at the inner side of the axis (B). Inserts with three-dimensional representation show two adjacent subunits of each oligomer, with the intersubunit axis relating rotation of adjacent subunits during transition between the two states shown in yellow.

There are only few cases where accurate structural information is available on alternative circular oligomers formed by chemically identical subunits. In addition to TRAP, X-ray structures of alternative states are available for the SAP-like pentraxin from *Limulus polyphemus* [25], for the protective antigen protein of *Bacillus anthracis* [7], [8] and for the small terminase from bacteriophage SF6 [Bűttner et al., to be published]. The SAP-like pentraxin exists as a natural mixture of 7-subunit and 8-subunit oligomers. Likewise, the SF6 small terminase appears to exist as a mixture of 9-subunit and 10-subunit oligomeric forms. In the case of the protective antigen however, the transition between 7-mer and 8-mer oligomers is controlled by the loop of residues 305–324 located at the outer side of the inter-subunit rotation axis, removal of this loop stabilizes the 8-mer state [8]. For all four proteins, the transition from one oligomeric state to another is achieved by a simple rotation of adjacent subunits around inter-subunit axes, as seen in TRAP. On the basis of these structural observations we propose a general mechanism for transition between different oligomeric states of a multisubunit circular protein, **Figure 5**, which involves rigid-body rotation around inter-subunit axes. These overall rotations are accompanied by minor conformational adjustments at subunit-subunit interfaces facilitated by proteins' plasticity. Notably, comparison of the alternative oligomeric states of all four proteins shows that the transition from an n-subunit to (n+1)-subunit state roughly doubles the seemingly obvious (n+1)/n increase in the diameter of the central tunnel, **Table 4**. This is because each inter-subunit rotation axis is positioned in the center of the subunit-subunit interface and not at its inner edge. This arrangement minimizes structural changes at the interfaces.

**Table 4:** Increase in the size of the central tunnel.

Protein	*D_n_*, diameter of the central tunnel (*D*, Å)	*D_n+1_*, diameter of the central tunnel (*D*, Å)	Scaling up factor	Observed ratio
	in the oligomer with *n* subunits	in the oligomer with (*n+1*) subunits	(n+1)/n	(*D_n+1_*)/*D_n_*
TRAP	26.6_11_	31.6_12_	1.09	1.19
				
Small	13.0_9_	15.5_10_	1.11	1.19
Terminase				
Protective	30.9_7_	40.4_8_	1.14	1.31
Antigen				
SAP-like	43.8_7_	55.0_8_	1.14	1.26
Pentraxin				

The diameters correspond to circles defined by the Cα atoms of residues located at the tunnel's surface. In the case of TRAP, the small terminase (Bűttner et al., to be published) and the SAP-like pentraxin [25], which are composed of compact subunits containing single domains, the indicated diameter corresponds to residues 7, 95 and 185, respectively. In the case of the protective antigen [7], which contains multiple domains per subunit, the indicated diameter corresponds to residue 473 located in the oligomerization part of domain 2 where subunits interact with each other.

### C-terminus defines the oligomerization state of TRAP

Comparison of the *B. halodurans* TRAP structure with the available structures of TRAP 11-mers indicated that the C-terminal segment consisting of residues 71–75 plays a critical role in selecting between 11-mer and 12-mer TRAP. The conformational change in the C-terminal residues of *B. halodurans* TRAP occurs owing to Asp71 forming a salt bridge with the side chain of Lys15, pulling the C-terminal residues 72–76 out of the subunit-subunit interface, **Figure 2D**; there is no such interaction in the 11-mer TRAP proteins since Lys15 is substituted by either Leu15 in *B. stearothermophilus* TRAP or Val15 in *B. subtilis* TRAP. Thr72 of *B. halodurans* TRAP also contributes to the diversion of the main chain. The carbonyl group of the corresponding residue, Ser72, in both *B. subtilis* and *B. stearothermophilus* TRAP form a hydrogen bond with the main chain of Ala61, fixing the position of the C-terminus next to the β-strands of neighboring subunit, **Figures 2B and C**. Such an interaction is prohibited in *B. halodurans* TRAP owing to steric hindrance caused by the methyl group of Thr72, thus facilitating the twisting of the main chain, **Figure 2D**. Sequence alignments show that, like *B. halodurans* TRAP, the TRAP proteins of *Paenibacillus sp.* and *Bacillus clausii* also have Lys at position 15 and Thr at position 72, **Figure 1A**. It is likely that these TRAP proteins could also exist as 12-mer assemblies.

It appears that the variation in oligomer number is a consequence of the evolutionary process. TRAP molecules that contain eleven subunits may represent a more evolved, minimized resource efficient version of a common ancestor. Alternatively, while the strength of the interaction between the RNA leader region and different TRAP molecules has been shown to be essentially identical, the potential influence of the oligomeric state on the interaction with other cellular factors cannot be excluded.

Artificial 12-mer TRAP molecules have been previously constructed by linking multiple *B. stearothermophilus* TRAP monomers into one continuous polypeptide chain [26]. Linking the C- and N-termini of three or four adjacent subunits with three-residue poly-alanine linkers resulted in 12-mers (called TRAP3 in the case of a three-subunit polypeptide). It was argued that entropy plays a major role in selecting the oligomeric state of TRAP, choosing a minimum number of separate polypeptide chains - 11 in the case of wild type TRAP and 4 in the case of TRAP3 [26]. However, structural comparisons and inspection of the electron density maps shows significant differences in conformation of C-terminal residues in the artificial 12-mer. In this molecule residue 72 is displaced from the subunit interface towards the oligomer's surface and there is no interpretable electron density for residue 73 suggesting it is flexible or has significant conformational variability. The altered conformation of the C-terminus in the artificial TRAP3 protein is apparently caused by the three-residue peptide linker (that links individual polypeptides) being simply too short to maintain the native configuration of the C-terminus. The conformational change in the artificial TRAP thus appears to parallel the behavior in the *B. halodurans* TRAP 12-mer, creating space at one side of the rotation axis and allowing a rotational adjustment between adjacent subunits. Consequently the true reason for the formation of the artificial TRAP3 12-mer assembly is the conformational difference in the C-terminus induced by addition of the polypeptide linkers. This is not to say however that entropic effects do not contribute to the stability of protein assembly.

### How to change the oligomeric state of a circular assembly?

To conclude, structural data on alternative oligomeric states of TRAP and other circular proteins show how essentially identical subunits could assembly into different oligomeric states. The similarity in their structural behavior is striking: the conformation of individual subunits in different oligomeric states remains largely unchanged with the exception of short segments that modulate inter-subunit surfaces. The transition is achieved by a simple rigid body rotation of adjacent subunits.

Structural information on *B.halodurans* TRAP suggested how such a transition could be induced by additions or deletions at inter-subunit surfaces. We tested this hypothesis by introducing mutations in 11-subunit TRAP, showing that residue deletion at the outer side of the inter-subunit axis or addition at the inner side, **Figure 4**, resulted in transition from an 11-subunit to 12-subunit state. This approach thus offers a promising route for controlling and alternating the subunit number of a circular protein and the size of its central tunnel, **Figure 5**. For some protein molecules, which use the central tunnel for their mechanism, change in the tunnel's diameter will obviously have functional implications.

## Materials and Methods

### Gene cloning, protein purification, crystallization and data collection

The engineered *B. subtilis* and *B. stearothermophilus* TRAP including point mutations and truncations were generated using the QuikChange kit (Stratagene, US) and a pET9a plasmid containing the wild type gene. All TRAP proteins were produced and purified as described previously [10]. Before crystallization, protein samples were transferred into solution containing 20 mM Tris (pH 8.5), 300 mM NaCl and purified by size-exclusion chromatography using Superdex 200 Column (GE healthcare, UK).

Crystallization was carried out at 18°C using hanging drop vapor diffusion. For crystallization, *B. halodurans* TRAP was transferred into solution containing 10 mM triethanolamine (pH 8.0), 100 mM NaCl, 15 mM L-tryptophan and concentrated to 30 mg/ml. The reservoir contained 100 mM Hepes (pH 7.5), 20 mM MgCl_2_ and 16% polyacrylic acid 5100 (v/v). *B. stearothermophilus* E71stop TRAP was crystallized using 50 mg/ml protein solution in 20 mM Tris (pH 8.5), 300 mM NaCl and 5 mM L-tryptophan. The reservoir contained 100 mM Hepes (pH 7.5), 200 mM MgCl_2_ and 30% iso-propanol (v/v). For crystallization, *B. subtilis* TRAP K71stop was transferred into solution containing 20 mM triethanolamine (pH 8.5), 100 mM NaCl and 15 mM L-tryptophan and concentrated to 26 mg/ml. The reservoir contained 100 mM Bis-Tris-Propane (pH 8.5), 0.2 M Na/K tartrate and 10% PEG 3350 (v/v). Protein crystals were frozen using solutions containing all the crystallization ingredients with addition of 20% glycerol (v/v). The X-ray data were collected at 120 K using synchrotron radiation. In the case of *B. halodurans* TRAP the data were collected at the Diamond Light Source station I24. For *B. stearothermophilus* E71stop TRAP and *B. subtilis* K71stop TRAP, the data were collected at the ESRF station ID14-2 and station ID14-4, respectively. Data were processed using HKL2000 [27], **Table 1**.

### Structure determination and refinement

All crystallographic calculations were carried out using the CCP4 program package [28]. The structures were solved by molecular replacement using MOLREP [29] with three adjacent subunits of *B. stearothermophilus* TRAP as a search model. Refinement was performed by REFMAC [30] and model rebuilding was done using COOT [31]. Water molecules were added automatically with the program ARP/wARP [32] and further corrected using maximum likelihood-weighted 2|F_o_| - |F_c_| and |F_o_| - |F_c_| electron density maps. Molecular contacts between adjacent monomers of TRAP were examined by CONTACT [28]. All figures were generated using CCP4mg [33].

### Native Mass spectrometry

All protein samples were in solution containing 100 mM ammonium acetate (pH 7.5) and 10 µM L-tryptophan at a concentration of 0.1–0.4 mg/ml. Mass spectrometry was performed using an orthogonal acceleration time-of-flight LCT premier XE system (Waters, MA, US), equipped with an offline nanoflow emitter (New Objective, MA, US). Mass spectra were acquired over the range 2000 to 8000 m/z, integrated over 5 sec intervals. Masslynx 4.1 software (Waters, MA, US) was employed to analyze the results. Molecular masses and standard deviations were calculated from the centroid values of species with at least three charge states. The data were calibrated externally with CsI solution (10 mg/ml). Measured masses (**Table 2**) are somewhat greater than calculated, as observed earlier for other systems [34].

## Supporting Information

Text S1 **Containing detailed description of L-Tryptophan binding sites.**
(DOCX)

Figure S1 **Ribbon diagrams of** ***B. stearothermophilus*** **TRAP E71stop (A),** ***B. subtilis*** **TRAP K71stop (B) and** ***B. halodurans*** **TRAP (C) viewed along the 12-fold axis.** Each subunit is shown in a different color. L-tryptophan molecules are shown as van der Waals models with carboxyl oxygen atoms in red, nitrogen atoms in blue and carbon atoms in yellow. In *B. halodurans* TRAP one additional L-tryptophan per monomer is bound at the surface close to the entrance into the central tunnel.
(DOC)

Figure S2 **Tryptophan binding.** Both *B. halodurans* TRAP (A) and *B. stearothermophilus* TRAP E71stop (B) bind tryptophan between adjacent subunits. *B. halodurans* TRAP contains additional tryptophan binding site (C) at the surface close to the entrance of the central tunnel. The 2mF_o_ - DF_c_ electron density maps are contoured at 1σ. Carbon atoms are shown in green, oxygen atoms in red, nitrogen atoms in blue, and sulfur atoms in yellow.
(DOC)

Table S1 **Main chain inter-subunit hydrogen bonding distances (Å) between the β-strand atoms in different TRAP oligomers.**
(DOCX)

Table S2 **Average inter-subunit hydrogen bonding distances in the wild type and E71stop** ***B. stearothermophilus*** **TRAP.**
(DOCX)

Table S3 **Average inter-subunit hydrogen bonding distances in wild type and K71stop** ***B. subtilis*** **TRAP proteins.**
(DOCX)
