# Supplementary material for: How to Change the Oligomeric State of a Circular Protein Assembly: Switch from 11-Subunit to 12-Subunit TRAP Suggests a General Mechanism
Source: PLoS One. 2011 Oct 3;6(10):e25296. doi: 10.1371/journal.pone.0025296 (PMC3184956; doi:10.1371/journal.pone.0025296)
Supplement: Text S1 — Containing detailed description of L-Tryptophan binding sites. (DOCX) [file pone.0025296.s001.docx]

**Supporting text S1**

**L-Tryptophan binding sites**

In *B. halodurans* TRAP, tryptophan (12 per oligomer) is bound with its side chain buried in a deep hydrophobic pocket constructed by two adjacent subunits, **Figure 1B**, and with its amino and carboxyl moieties forming an invariant and extensive network of hydrogen bonding interactions, **Figures S2A, B.** As in *B.subtilis* TRAP [1, 2], most hydrogen bonding interactions are with residues in surface loops 25-33 and 49-52. The 11-mer and 12-mer TRAP proteins differ by two amino acid substitutions within the tryptophan binding pocket. In *B. stearothermophilus* TRAP, Leu24 interacts with Leu44 from the neighboring chain stabilizing the hydrophobic cluster, while residues Leu24 and Ile44 play the same role in *B. subtilis* TRAP. In *B. halodurans* TRAP the corresponding residues are Met24 and Met44, respectively, **Figure S2A**. These substitutions do not cause any significant conformational adjustments in surrounding residues as the two residues serve to fill the hydrophobic pocket around the indole. Apart from the buried L-tryptophan molecules bound between neighboring subunits, the structure of *B. halodurans* TRAP contains one additional L-tryptophan per monomer bound at the surface close to the entrance of the central tunnel, **Figure S1C**. This tryptophan molecule is exposed at the surface of TRAP making only two hydrogen bonding interactions with the protein, **Figure S2C**, suggesting that this is a low-affinity binding site occupied owing to the high concentration of tryptophan present in crystallization. Indeed, native mass spectrometry with samples containing 10 μM L-tryptophan did not detect species with more than one tryptophan molecule per subunit of TRAP, **Figure 4A** and **Table 2**.

**References:**

1. Antson AA, Otridge J, Brzozowski AM, Dodson EJ, Dodson GG, Wilson KS, Smith TM, Yang M, Kurecki T, Gollnick, P. (1995) The structure of *trp* RNA-binding attenuation protein. Nature 374: 693–700.
2. Yakhnin AV, Trimble JJ, Chiaro CR, Babitzke P (2000) Effects of mutations in the L-tryptophan binding pocket of the *trp* RNA-binding attenuation protein of *Bacillus subtilis*. J Biol Chem 275: 4519-4524.
