# Supplementary material for: How to Change the Oligomeric State of a Circular Protein Assembly: Switch from 11-Subunit to 12-Subunit TRAP Suggests a General Mechanism
Source: PLoS One. 2011 Oct 3;6(10):e25296. doi: 10.1371/journal.pone.0025296 (PMC3184956; doi:10.1371/journal.pone.0025296)
Supplement: Figure S1 — Ribbon diagrams of B. stearothermophilus TRAP E71stop (A), B. subtilis TRAP K71stop (B) and B. halodurans TRAP (C) viewed along the 12-fold axis. Each subunit is shown in a different color. L-tryptophan molecules are shown as van der Waals models with carboxyl oxygen atoms in red, nitrogen atoms in blue and carbon atoms in yellow. In B. halodurans TRAP one additional L-tryptophan per monomer is bound at the surface close to the entrance into the central tunnel. (DOC) [file pone.0025296.s002.doc]

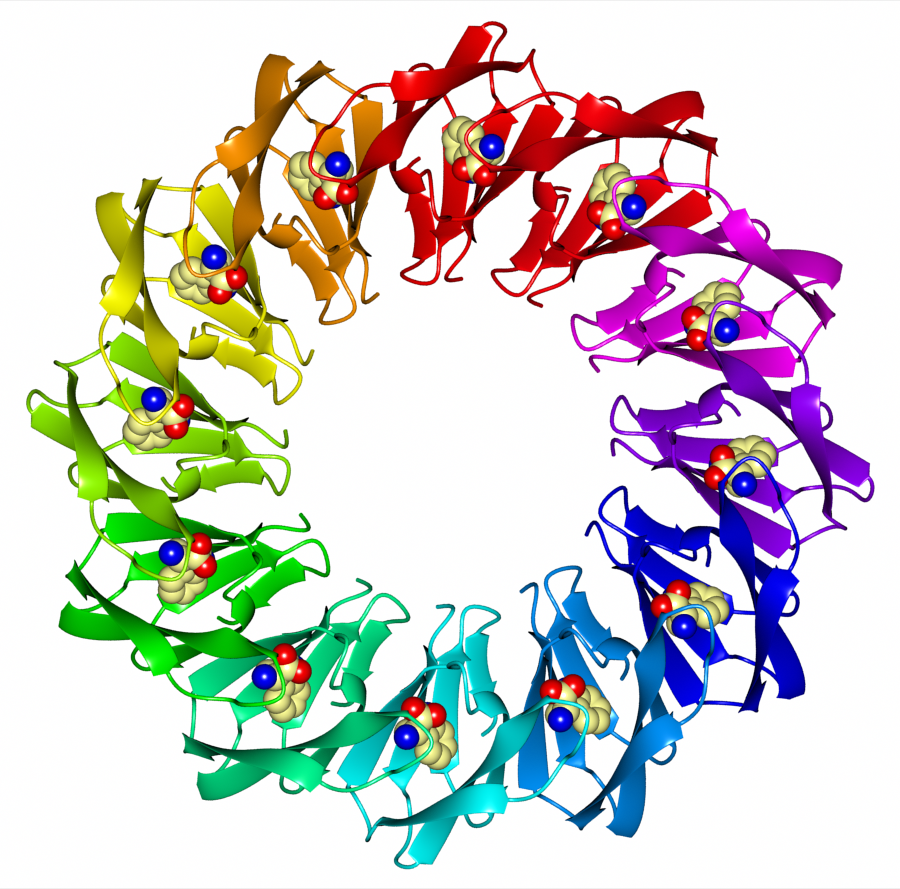

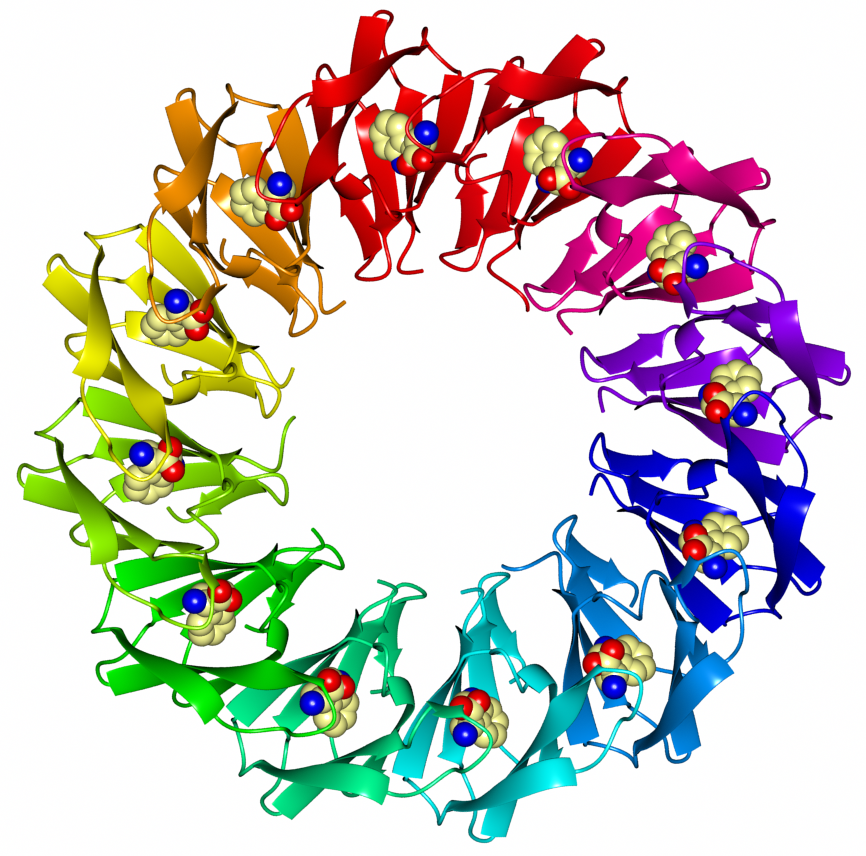


B

A

C


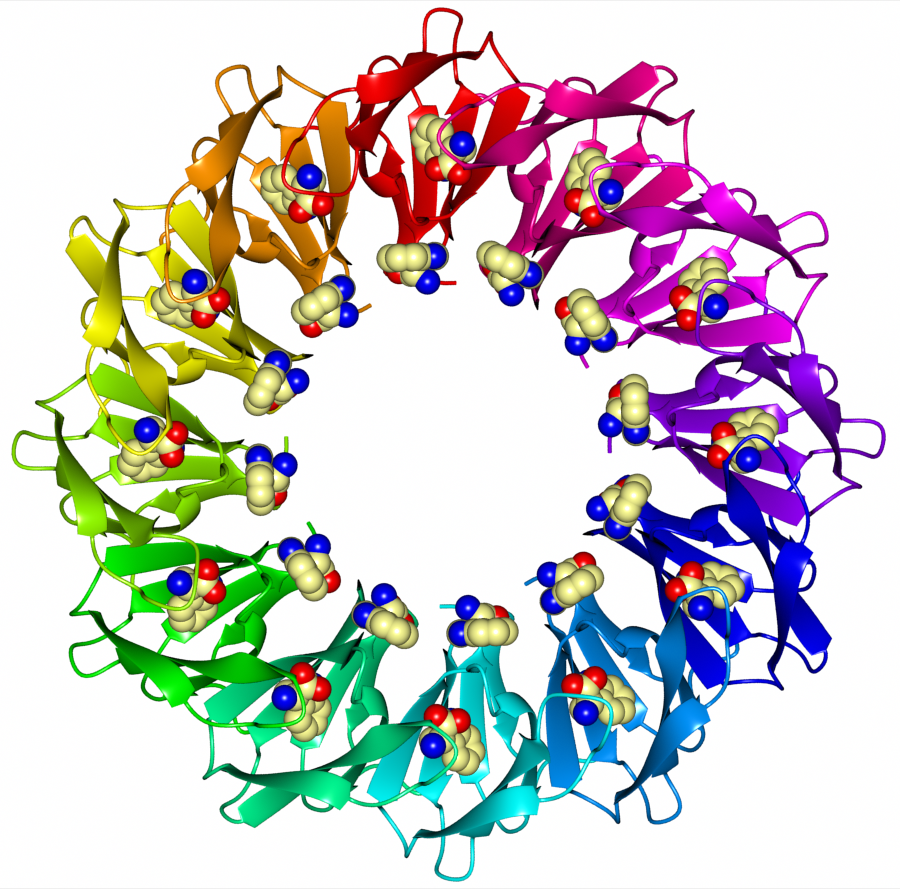


**Figure S1.** Ribbon diagrams of *B. stearothermophilus* TRAP E71stop (A), *B. subtilis* TRAP K71stop (B) and *B. halodurans* TRAP (C) viewed along the 12-fold axis. Each subunit is shown in a different color. L-tryptophan molecules are shown as van der Waals models with carboxyl oxygen atoms in red, nitrogen atoms in blue and carbon atoms in yellow. In *B. halodurans* TRAP one additional L-tryptophan per monomer is bound at the surface close to the entrance into the central tunnel.
