# Supplementary material for: How to Change the Oligomeric State of a Circular Protein Assembly: Switch from 11-Subunit to 12-Subunit TRAP Suggests a General Mechanism
Source: PLoS One. 2011 Oct 3;6(10):e25296. doi: 10.1371/journal.pone.0025296 (PMC3184956; doi:10.1371/journal.pone.0025296)
Supplement: Figure S2 — Tryptophan binding. Both B. halodurans TRAP (A) and B. stearothermophilus TRAP E71stop (B) bind tryptophan between adjacent subunits. B. halodurans TRAP contains additional tryptophan binding site (C) at the surface close to the entrance of the central tunnel. The 2mFo - DFc electron density maps are contoured at 1σ. Carbon atoms are shown in green, oxygen atoms in red, nitrogen atoms in blue, and sulfur atoms in yellow. (DOC) [file pone.0025296.s003.doc]

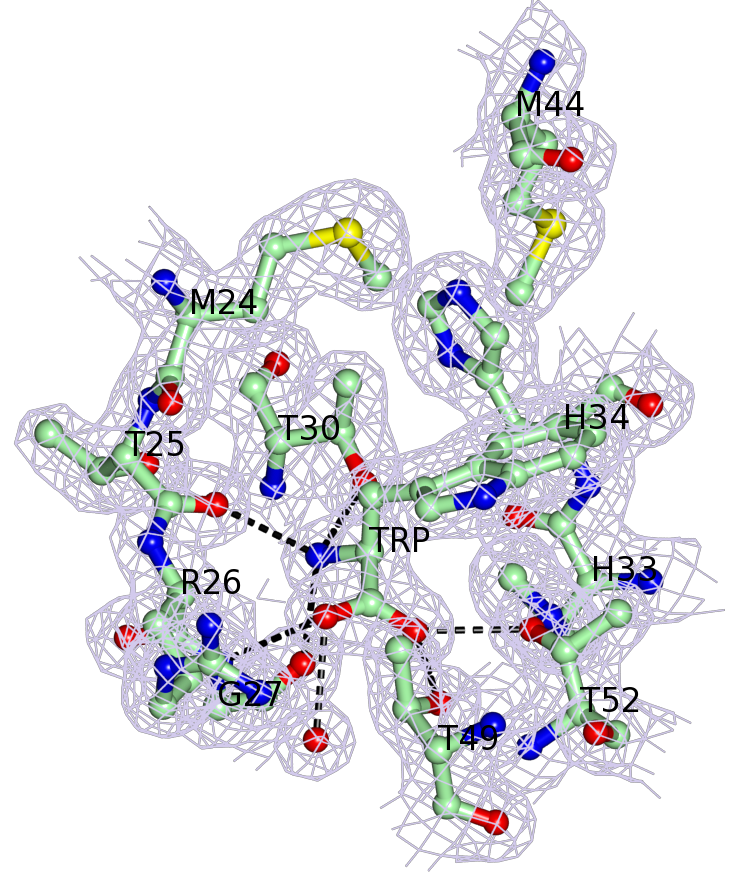

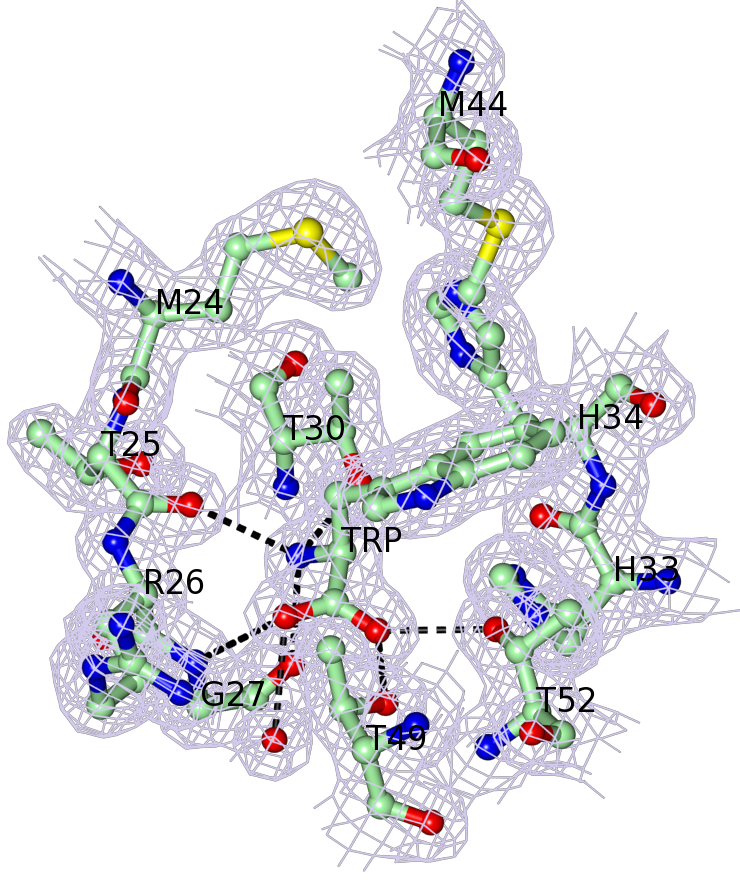


A


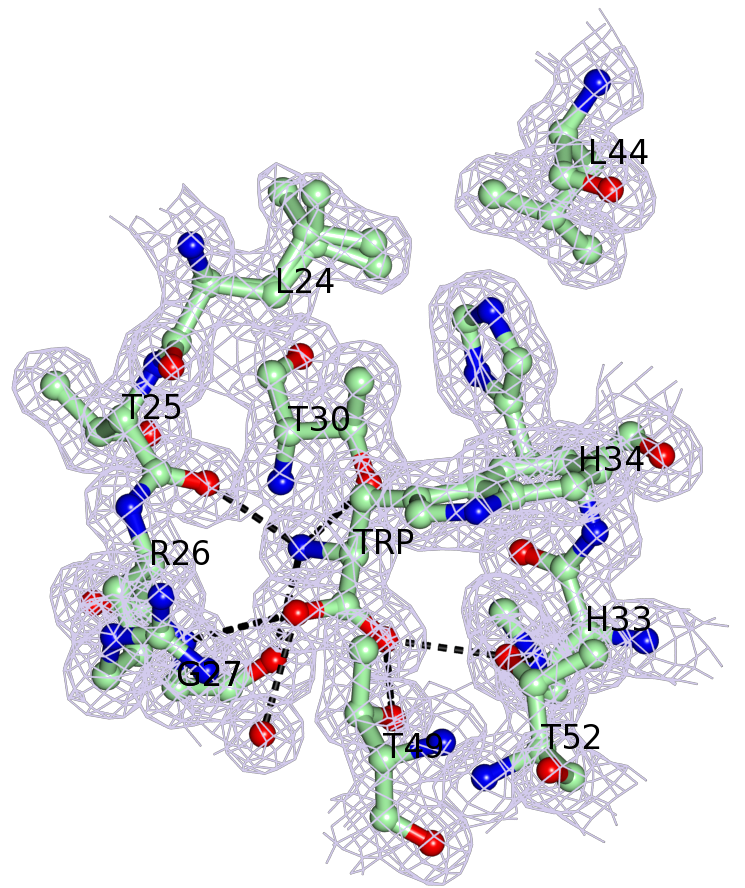

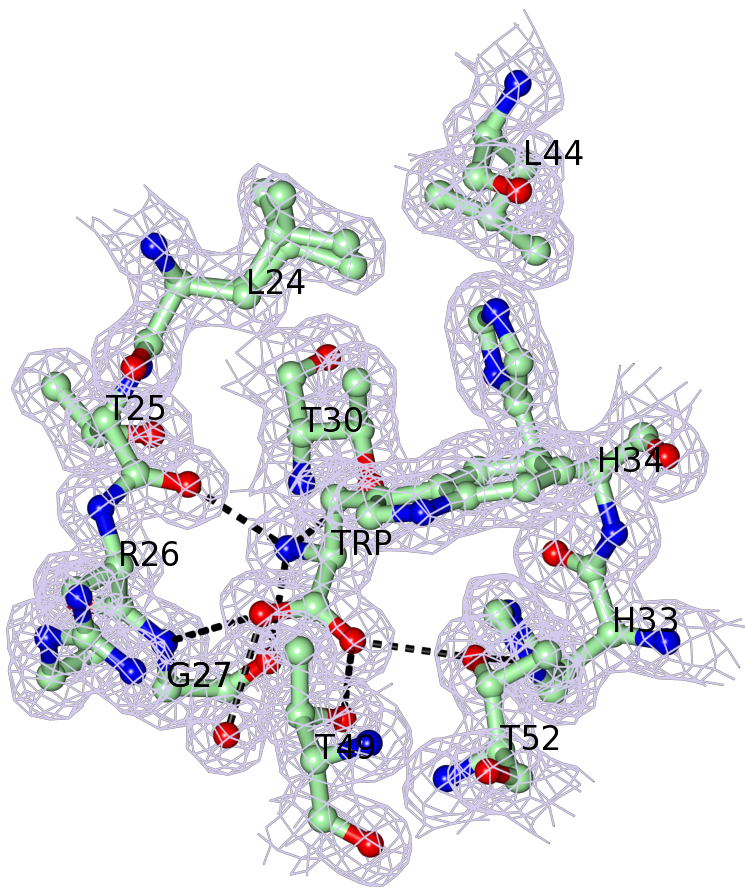


B

C


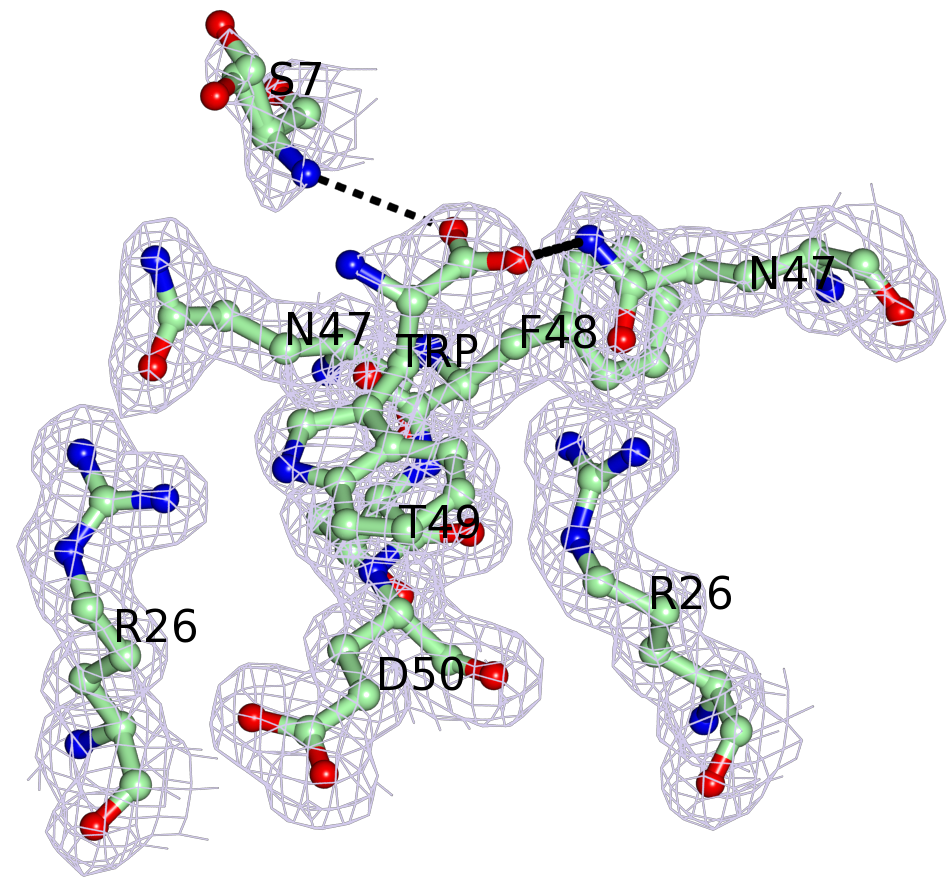

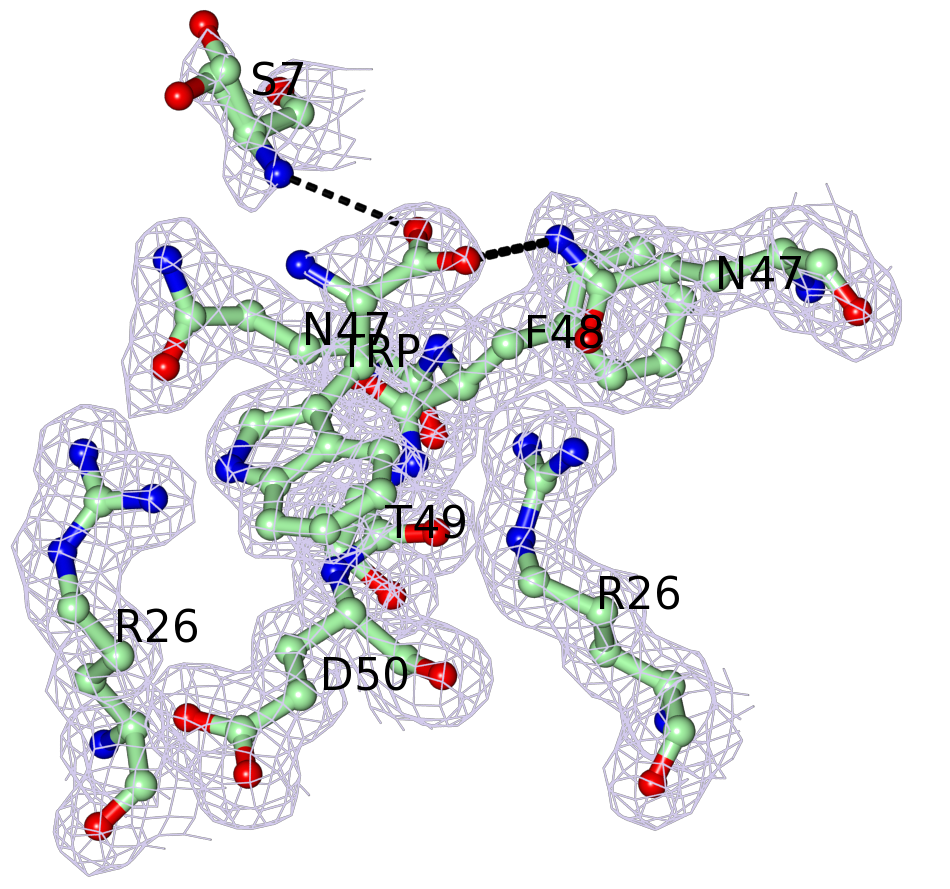


**Figure S2.** Tryptophan binding. Both *B. halodurans* TRAP (A) and *B. stearothermophilus* TRAP E71stop (B) bind tryptophan between adjacent subunits. *B. halodurans* TRAP contains additional tryptophan binding site (C) at the surface close to the entrance of the central tunnel. The 2mF_o_ - DF_c_ electron density maps are contoured at 1 σ. Carbon atoms are shown in green, oxygen atoms in red, nitrogen atoms in blue, and sulphur atoms in yellow.
