# Supplementary material for: How to Change the Oligomeric State of a Circular Protein Assembly: Switch from 11-Subunit to 12-Subunit TRAP Suggests a General Mechanism
Source: PLoS One. 2011 Oct 3;6(10):e25296. doi: 10.1371/journal.pone.0025296 (PMC3184956; doi:10.1371/journal.pone.0025296)
Supplement: Table S1 — Main chain inter-subunit hydrogen bonding distances (Å) between the β-strand atoms in different TRAP oligomers. (DOCX) [file pone.0025296.s004.docx]

**Table S1.** Main chain inter-subunit hydrogen bonding distances (Å) between the β-strand atoms in different TRAP oligomers.

| Atom in | Atom in | *B. halodurans* | Wild type | K71stop | Wild typ | E71stop |
| --- | --- | --- | --- | --- | --- | --- |
| chain A | chain B | TRAP | *B. subtilis* | *B. subtilis* | *B. stearo* | *B. stearo* |
|  |  |  | TRAP | *TRAP* | *TRAP* | *TRAP* |
| S53O | Q47N | 3.02-3.10 | 2.96-3.13 | 3.01-3.05 | 3.06-3.20 | 3.02-.3.04 |
|  |  | (3.05) | (3.03) | (3.03) | (3.13) | (3.03) |
| V55N | I45O | 2.83-3.07 | 2.77-2.93 | 2.82-2.86 | 2.69-2.99 | 2.81-2.86 |
|  |  | (2.92) | (2.84) | (2.84) | (2.85) | (2.83) |
| V55O | I45N | 2.99-3.00 | 2.67-2.87 | 2.81-3.05 | 2.68-3.01 | 2.77-2.82 |
|  |  | (2.99) | (2.77) | (2.87) | (2.83) | (2.79) |
| I57N | V43O | 2.75-2.78 | 2.76-2.87 | 2.78-2.82 | 2.71-3.14 | 2.77-2.82 |
|  |  | (2.76) | (2.82) | (2.80) | (2.96) | (2.79) |
| I57O | V43N | 2.99-3.09 | 3.03-3.20 | 3.00-3.10 | 3.23-3.92 | 3.03-3.08 |
|  |  | (3.05) | (3.15) | (3.04) | (3.53) | (3.06) |

Atoms are labeled by single letter amino acid code, residue number and symbol. Residue numbering is as in *B. subtilis* TRAP. Minimum, maximum distances as well as distances averaged over 11 (or 12) interfaces are shown. *B. stearo – B. stearothermophilus*.
