# Supplementary material for: How to Change the Oligomeric State of a Circular Protein Assembly: Switch from 11-Subunit to 12-Subunit TRAP Suggests a General Mechanism
Source: PLoS One. 2011 Oct 3;6(10):e25296. doi: 10.1371/journal.pone.0025296 (PMC3184956; doi:10.1371/journal.pone.0025296)
Supplement: Table S2 — Average inter-subunit hydrogen bonding distances in the wild type and E71stop B. stearothermophilus TRAP. (DOCX) [file pone.0025296.s005.docx]

**Table S2.** Average inter-subunit hydrogen bonding distances in the wild type and E71stop *B. stearothermophilus* TRAP.

| Atoms in | Atoms in | Average distance | Average distance |
| --- | --- | --- | --- |
| chain A | chain B | (Å) in wild type | (Å) in E71stop |
|  |  | TRAP | TRAP |
| E71O | K13Nζ | 2.82 ± 0.24 | 2.73 ± 0.05 |
| K56Nξ | E36Oε2 | 2.67 ± 0.22 | 2.75 ± 0.04 |
| K56Nξ | K37O | 2.89 ± 0.11 | 2.93 ± 0.14 |
| R58Nε | E42Oε2 | 2.94 ± 0.23 | 2.98 ± 0.16 |
| V57N | V43O | 2.96 ± 0.13 | 2.79 ± 0.02 |
| I55N | I45O | 2.85 ± 0.12 | 2.83 ± 0.02 |
| I55O | I45N | 2.83 ± 0.08 | 2.79 ± 0.02 |
| R26NH2 | Q47Oε1 | 2.78 ± 0.22 | 2.74 ± 0.01 |
| S53O | Q47N | 3.13 ± 0.06 | 3.03 ± 0.01 |
| V69O | E64Nε2 | 3.03 ± 0.13 | 3.03 ± 0.04 |
| V57O | V43N | 3.53 ± 0.24 | 3.06 ± 0.02 |
| E73N | G41O | 2.83 ± 0.19 | N/A |
| H67Nε2 | F9O | 3.07 ± 0.01 | 3.14 ± 0.14 |

Atoms are labeled by single letter amino acid code, residue number and symbol. Hydrogen bond lengths, averaged over 11 (wild type TRAP) or 12 (E71stop TRAP) subunits are shown with a cut-off distance of 3.2 Å. N/A- not applicable in the case of E71stop TRAP.
