# Supplementary material for: How to Change the Oligomeric State of a Circular Protein Assembly: Switch from 11-Subunit to 12-Subunit TRAP Suggests a General Mechanism
Source: PLoS One. 2011 Oct 3;6(10):e25296. doi: 10.1371/journal.pone.0025296 (PMC3184956; doi:10.1371/journal.pone.0025296)
Supplement: Table S3 — Average inter-subunit hydrogen bonding distances in wild type and K71stop B. subtilis TRAP proteins. (DOCX) [file pone.0025296.s006.docx]

**Table S3.** Average inter-subunit hydrogen bonding distances in wild type and K71stop *B. subtilis* TRAP.

| Atoms in | Atoms in | Average distance (Å) | Average distance (Å) |
| --- | --- | --- | --- |
| chain A | chain B | in wild type TRAP | in K71stop TRAP |
| Y67OH | F9O | 2.83 ± 0.24 | 3.22 ± 0.03 |
| Y67OH | D08Oδ1 | 2.82 ± 0.25 | 3.80 ± 0.07 |
| E73Oε2 | K13Nζ | 2.60 ± 0.18 | N/A |
| K56Nζ | E36Oε2 | 2.78 ± 0.15 | 2.76 ± 0.08 |
| K56Nζ | K37O | 2.91 ± 0.18 | 2.95 ± 0.11 |
| E73N | G41O | 2.87 ± 0.09 | N/A |
| V57N | V43O | 2.82 ± 0.03 | 2.80 ± 0.01 |
| V57O | V43N | 3.15 ± 0.06 | 3.04 ± 0.05 |
| I55N | I45O | 2.84 ± 0.05 | 2.84 ± 0.01 |
| I55O | I45N | 2.77 ± 0.07 | 2.83 ± 0.02 |
| S53O | Q47N | 3.03 ± 0.05 | 3.03 ± 0.02 |
| R26NH2 | Q47Oε1 | 2.82 ± 0.08 | 2.74 ± 0.02 |
| R26NH1 | E50Oε1 | 3.00 ± 0.06 | N/A |
| E69O | Q64Nε2 | 3.01 ± 0.09 | 2.98 ± 0.12 |

Atoms are labeled by single letter amino acid code, residue number and symbol. Hydrogen bond lengths, averaged over 11 (wild type TRAP) or 12 (K71stop TRAP) subunits are shown with a cut-off distance of 3.2 Å. N/A- not applicable in the case of K71stop TRAP.
